# Identification and evaluation of the core elements of character education for medical students in Korea

**DOI:** 10.3352/jeehp.2019.16.21

**Published:** 2019-08-20

**Authors:** Yera Hur, Keumho Lee

**Affiliations:** 1 Institute of Medical Education, Hallym University College of Medicine, Chuncheon, Korea; 2 Korea Institute for Research in the Behavioral Sciences, Seoul, Korea; The Catholic University of Korea, Korea

**Keywords:** Character, Medical student, Medical education, Delphi technique, Korea

## Abstract

**Purpose:**

Medicine requires uniquely high levels of motivation, ethics, and altruistic values and behavior. This study was conducted to redefine character education in medical education and to identify and evaluate the core elements of physicians’ character.

**Methods:**

A 3-round Delphi survey was conducted among professors of medical education, physicians, experts from nursing schools, and a head nurse in Korea. A consultant group (CG) was formed to prepare the Delphi survey, discuss the research results, and set directions for future initiatives. The 3 rounds of the Delphi survey were conducted between September 2018 and February 2019.

**Results:**

From the first-round Delphi survey, which inquired about the 10 key character elements required for medical students, a total of 420 elements were collected. The top 10 categories were selected and classified. After the second and third rounds of the Delphi consensus process and a series of CG meetings, the following 8 core categorical elements were identified: service and sacrifice, empathy and communication, care and respect, honesty and humility, responsibility and calling, collaboration and magnanimity, creativity and positivity, and patience and leadership. The average score of medical graduates for the core elements ranged from 2.45 to 3.46 (standard deviation, 0.23–0.60) on a 5-point Likert scale.

**Conclusion:**

Eight core categorical elements of the character of medical students were identified. The results of this study can be used as a reference for establishing the goals and desired outcomes of character education at the level of undergraduate or graduate medical education.

## Introduction

According to a survey conducted by the Korean Education Development Institute (2014), 72.4% of Koreans said that the level of morality and personal character of Korean students is generally low, and that character education is the most urgent issue that should be addressed [1]. In other words, Korean students are not adequately equipped with mutual respect, consideration, and honesty in human relations. Many medical schools try to select students with a good character by using personality inventories or interviews to inform admissions decisions [2,3]. Some Korean medical schools select students using academic achievement as the top priority. A consequence of this system is that students with low levels of morality or a questionable character can enter medical schools.

Doctors are required to have a higher level of character and professionalism than other professionals, with important factors including high vocational consciousness, ethical standards, and altruistic values [4]. Thus, it is necessary to assess the personality or character of applicants to medical school, but doing so is not easy within the Korean entrance examination system. For this reason, medical schools have made efforts to provide character education to medical students through medical humanities and social medicine curricula emphasizing communication skills, medical ethics, and community service [5]. Nevertheless, within medical schools, unethical behaviors such as sexual harassment, kickbacks, and cheating continue to occur. The negative public reaction to such behaviors has made it necessary to examine the current process of character education in medical schools and to propose some alternatives.

Although numerous previous studies have shared experiences with curricula on medical professionalism, medical ethics, community services, medical humanities, and communication skills, and have addressed the necessity of character education [6-10], it is difficult to find studies directly dealing with the current problems in character education and suggesting alternatives for medical education. In order for medical students to develop an identity as a doctor, their academic, clinical, and patient-related competencies are all important, and these 3 factors must be well-balanced [11]. With this in mind, it is necessary to examine whether the current medical education system is appropriate for producing good doctors, and in particular, whether character education is included along with medical knowledge and skills. For the purposes of this study, character education means developing ethical/behavioral values, personality, virtue, and a human image suitable for becoming a good doctor. Although it is difficult to summarize the definition or qualities of a good doctor in a single word, this study approached the character aspects that good doctors should have.

It is necessary to distinguish character from professional clinical performance, because while some aspects of character overlap with the complex concepts of medical professionalism and the medical humanities, which include all the knowledge, skills, and attitudes required by good doctors, it is more desirable to view character as a distinct phenomenon. Therefore, this study aimed to evaluate and redefine character education in Korean medical schools by investigating the following questions: (1) What are the core elements of character that good doctors should have?; (2) How can we define those core elements, and what sub-elements do they contain?; (3) How can a model of character education for medical students be presented?; (4) What are medical students’ levels of the identified core elements?; and (5) What is the appropriate academic year during which the core elements should be provided?

## Methods

### Ethical statement

All participants participated in the survey after providing written informed consent. This study was approved by the Institutional Review Board of Hallym University (HIRB-2018-049).

### Study design

**Fig. 1.**
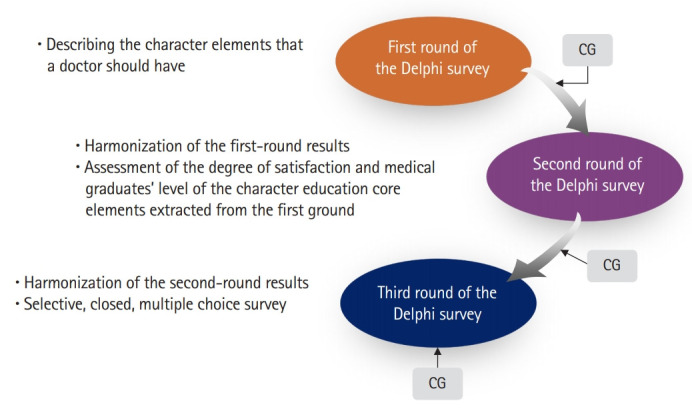
Study design of the 3-round Delphi survey. CG, consultant group.

In order to identify the key character elements that doctors should have and to create a character education model for Korean doctors, we conducted a literature review and case analysis. Based on this, a 3-round Delphi survey and consultant group (CG) meetings were conducted (Fig. 1).

The participants of the Delphi survey were Korean professors of medical education, physicians, experts from nursing schools, and a head nurse. The first round of the Delphi survey consisted of 5 open questions: the necessity of character education in medical education, the personal character required by doctors, what is considered “character,” the problems and failures of character education in current medical education, and the key character elements for doctors in the fourth industrial age. Participants were asked to list 10 or more key elements, numbered from 1 to 10 in order of importance.

The second round of the Delphi survey was conducted among respondents who participated in the first round. The questions focused on assessing respondents’ degree of satisfaction with the core elements of character education extracted from the first round and with the level of graduates regarding those skills. The respondents could also suggest corrections or deletions of the core elements, and provide additional comments. The third round of the Delphi survey was only conducted among respondents who reported discordant responses in the second round of the Delphi analysis, and consensus on the second-round responses was reached.

### Materials and subjects

For the Delphi survey, the manual for Delphi participants and consent form were distributed together. The content included the background and purpose of the research, subjects, research methods, duration of the research, a statement that participants could withdraw from the study, the disadvantages if the study was not done, personal information, and a statement on confidentiality.

To create the Delphi questionnaire, discuss the results of the rounds of the Delphi survey, and set directions for future initiatives, a CG was assembled with a total of 17 participants, including 2 character education specialists, 3 medical education professors, 2 professors from nursing school, 2 physicians, 2 members of the general public, 1 head nurse, and 6 medical students. Some of them (3 medical education professors, 2 physicians) also participated in the Delphi survey. The questionnaires used are presented in the Appendix 1.

The medical education experts selected for the Delphi survey were drawn from professors of medical education at 40 medical schools in Korea, to ensure the greatest possible representativeness. The medical education experts were also selected from the past and present board members of the 2 major representative institutions leading medical education in Korea, the Korean Society of Medical Education and the Korean Institute of Medical Education and Evaluation. Therefore, 67 medical professors and 63 members of the institute were selected for the Delphi survey. Eleven physicians and 2 experts from nursing schools were added to the list based on suggestions from the CG. A total of 143 subjects were selected for the Delphi survey. The number of Delphi experts is generally between 30 and 100; however, a higher number was chosen given the busy schedules of medical professors and to prepare for possible drop-out of participants during the multiple survey rounds. Three rounds of the Delphi survey were conducted between September 2018 and February 2019. In the first round of the Delphi survey, 47 (32.9%) of the 144 invited subjects replied. In the second round of the Delphi survey, 38 (80.8%) of the 47 respondents in the first round of the Delphi survey participated. In the third round of the Delphi survey, only 26 respondents participated, corresponding to 100% of those who disagreed in the second survey (Table 1).

### Statistics

Descriptive statistics were calculated, such as the frequency, mean, and standard deviation.

## Results

### The necessity and problems of character education in medical education

The first round of the Delphi survey asked open questions about the necessity and problems of character education in medical education. There was 1 non-response, and all 46 respondents stated that character education was essential in medical education. Regarding the problem of character education, there was a considerable number of opinions based on factors such as a knowledge-oriented educational system, lack of an appropriate curriculum, lack of concern with individuals’ character as an evaluation factor in the course of becoming a doctor, inadequate awareness of the need for character education among professors and students, and insufficient teaching ability to handle character education properly.

Some respondents also expressed the opinions that the concept of character is ambiguous, and that there is a lack of research on what content to teach. In particular, many respondents pointed out that medical school professors or students do not value character education, but only academic performance. The raw data are available in Supplement 1.

### The definition of character education

In order to define the concept of character required by doctors, this question was included in the first round of the Delphi survey. Forty-seven respondents gave a variety of comments, which could be summarized as follows. The character that a doctor requires is the basic attitude, values, and mindset that must be present to perform his or her duties. These include respect for human beings, empathy and consideration for patients, a sense of calling, honesty, ethics, and responsibility.

### Identification of character education core elements, and the sub-elements

In the first round of the Delphi survey, a total of 420 elements were collected from respondents’ lists of the 10 key elements required for medical students. The content of each element was analyzed, and similar elements were grouped into 17 items, the top 10 of which were selected for inclusion in the major classification.

After the second and third rounds of the Delphi survey and the CG meeting, we were able to identify 8 core categories of character education: service and sacrifice, empathy and communication, care and respect, honesty and humility, responsibility and calling, collaboration and magnanimity, creativity and positivity, and patience and leadership. These core elements, together with their sub-elements, are presented in Table 2.

### The model of character education for medical students

**Fig. 2.**
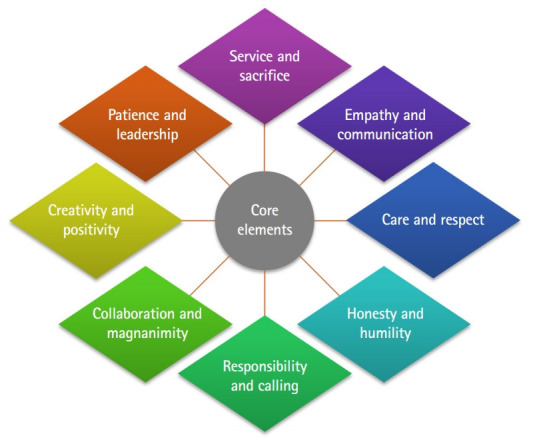
The 8 core categorical elements of character education.

Fig. 2. shows the 8 core categorical elements for the character education of medical students derived from the Delphi survey and the CG. The sub-elements with similar concepts were integrated and the final sub-elements were selected to fit the 8 core categorical elements, and their definitions are shown in Table 3. The final names and definitions of the 8 core categorical elements and the sub-factors of each core element were selected through expert meetings.

### Medical students’ level of the core elements

**Fig. 3.**
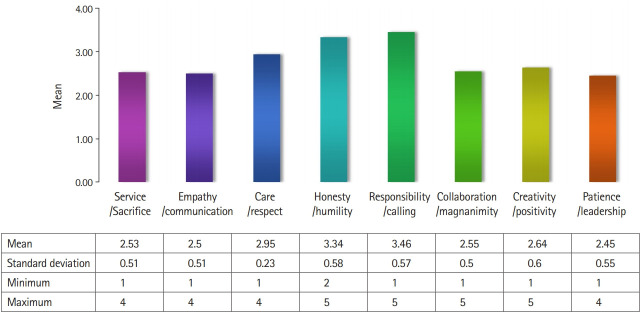
Evaluation of medical school graduates’ levels of the 8 core categorical elements of character.

In the third round of the Delphi survey, medical graduates’ levels of the 8 categorical core elements were rated on a scale ranging from very low (1 point) to very high (5 points) (Fig. 3). The average level of graduates for the 8 categorical core elements was 2.45–3.46. The core factors that were rated highest were ‘responsibility and calling’ (mean=3.46, standard deviation=0.57), while ‘patience and leadership’ (mean=2.45, standard deviation=0.55) was the lowest.

### The appropriate academic year in which the core elements should be provided

The appropriate academic year for instruction on the 8 core categorical elements was investigated, and the results are shown in Table 4. Many respondents indicated that the core elements could be instructed not in any particular year, but in any or all academic years of the medical education process.

The majority of the respondents said that ‘service and sacrifice’ are necessary throughout the premedical years (n=31) and that ‘honesty and service’ are appropriate for all years of medical education (n=24). Many also said that ‘honesty and humility’ and ‘patience and leadership’ would be appropriate to focus on in the senior years of medical education, with the general opinion that education on all 8 core elements should be available in all years.

## Discussion

Discussions of the qualities of good doctors and the need for personality education in medical education can be found in several previous studies [6,7,12]. Nonetheless, the overall consensus in the literature is that character education in medical school has not been successful, and the current problems of character education need to be reviewed. In order for character education to be successful, it is necessary to reach consensus on the core human factors desirable for medical students. Therefore, this study aimed to redefine character education in medical education using the Delphi technique and to identify key elements of character education for future doctors. The key elements of character education found in this study are similar to those of medical professionalism, but medical professionalism differs in that it emphasizes not only elements of character, but also the knowledge and skills required in medical education [13,14].

### Limitations

Some of the respondents to the first round of the Delphi survey failed to respond to the second round, resulting in a decrease in the number of subjects. Although the Delphi survey itself was conducted among a small number of specialists, the loss of 9 of the 47 primary Delphi respondents could be a limitation for generalizing the results of the study. In particular, only a model of the key factors of character education for medical students was developed. Furthermore, research was not conducted on educational content or methods that could be used at actual instructional sites.

In the future, it will be possible to develop various research initiatives and programs using the core factor model of personality education developed in this study. Our suggestions for follow-up research and applications are as follows:

### Provision of basic guidelines for character education

When developing and implementing medical education, medical schools refer to standardized guidelines, such as “Learning outcomes of basic medical education” issued by the Korean Association of Medical Colleges [15]. Although each university has certain goals and objectives, it is necessary to include specific levels of education and essential content in order for students to qualify for the medical licensure examination and for medical schools to receive accreditation. It is expected that this research will provide basic guidelines for character education by identifying the core competencies of character that are essential for future doctors, providing a basis for each university to develop specific character education courses suitable for that institution.

### A basis for related research

This study provides a basis for diverse related studies. For example, if core competencies for character education are identified, a variety of educational content could be developed for the competency of “altruism” alone. In other words, since educational content, instructional methods, and evaluation methods may vary depending on the core competencies of character education, it will be possible to investigate which method is more suitable and to examine their effects when such programs are actually implemented. The study investigated the level of these competencies in graduates and the appropriate academic level of education for each core competency, and further studies could be conducted among more faculty members of medical schools, physician groups, and patient groups. By doing so, it will be possible to determine which core competencies are lacking among medical school graduates, to investigate when it is appropriate to provide education on those competencies, and to use those findings to develop character education programs.

### Development of a character education mentoring module for medical students

Mentoring methods for character education can be considered, because mentoring has already been found to be a desirable educational methodology for fostering personality traits, community awareness, a sense of responsibility, service spirit, interpersonal skills, and moral reflection, while bringing about overall growth [16]. Under the Character Education Promotion Act in Korea [17], personality education is emphasized for college students, as well as for students in primary and secondary schools, and various character education programs have been developed. An analysis of the literature on medical humanities curriculum for college students in Korea [18] found that the main components were self-understanding and socializing, emotional control and self-esteem, and connection with others.

However, medical school students have different personality traits from those required by general college students. For example, among the factors found in this study, respect for life and vocational consciousness were identified as personality traits that are more important for medical students than for general college students. Therefore, the program developed is not suitable for general college students.

In conclusion, based on the core elements of character education identified in this study, we expect to be able to develop character education programs or mentoring modules suitable for medical students.

## Figures and Tables

**Table 1.** Number of subjects and their affiliations in each round of the Delphi survey

Affiliations	Subjects of study	1st Delphi survey respondents	2nd Delphi survey respondents	3rd Delphi survey respondents
Medical professors^a)^	67 (46.9)	18 (38.3)	16 (42.1)	12 (46.2)
Institute^b)^	63 (44.0)	16 (34.0)	13 (34.2)	10 (38.5)
Physicians	11 (7.7)	11 (23.4)	7 (18.4)	3 (11.5)
Nursing professors	2 (1.4)	2 (4.3)	2 (5.3)	1 (3.8)
Total	143 (100.0)	47 (100.0)	38 (100.0)	26 (100.0)

Values are presented as number (%).

a) Some of the subjects had duplicated affiliations, which were included here.

b) Institute: Korean Society of Medical Education, Korean Institute of Medical Education and Evaluation.

**Table 2.** Character education core elements and sub-elements based on the survey results

Core elements	Sub-elements
Service and sacrifice	Service, sacrifice, dedication, sympathy, compassion, devotion, altruistic attitude, warmth, willingness, diligence, concession, fraternity, appreciation
Empathy and communication	Communication skills, empathy, communication orientation, conflict management, listening, sincerity, flexibility, sociality, humor, healthy interpersonal relationships, expressive power, a warm smile, consideration, compassion, expressing one’s thoughts in writing, patience
Care and respect	Consideration, respect, understanding of others (including patients), kindness, tolerance, sense of companion, understanding of diversity, respect for people, respect for themselves, pride, respect for life, love, sense of life ethics, understanding death, human nature, courtesy
Honesty and humility	Honesty, diligence, humility, ethical judgment, morality, conscience, moral judgment, spirit of compliance, integrity, truth, authenticity, seriousness, fairness, accuracy, reflection, compliance with principles, intellectual self-confidence, professional ethics
Responsibility and calling	Responsibility, involvement, commitment, accountability, value internalization, values, medical law, medical ethics, professional ethics, confidentiality, compliance, attitude of lifelong learning
Collaboration and magnanimity	Cooperation, an embracing spirit, community consciousness, collaboration, peer collaboration, teamwork, mutual exchange, interdependence
Creativity and positivity	Creativity, positivity, insight, judgment, critical thinking, decision, open-mindedness, creative thinking, mindfulness of looking at problems from multiple angles, imagination, courage, calm, passion
Patience and leadership	Leadership, challenging spirit, followership, leading, social problem consciousness, social cognitive ability, initiative, self-regulation, management, self-understanding, self-reflection, self-control, self-identity, capability, self-control, patience, well-being, balance, self-empathy, manners, courtesy, elegance, dignity, self-management

**Table 3.** Definition of the core elements of character education

Core elements	Definition
Service and sacrifice	Attitude of thinking of others (patients) before one’s own personal interests, sacrificing oneself for others, devoting oneself to society, and practicing volunteer work through medical practice
Empathy and communication	Attitude and ability to interact and communicate well while accurately communicating thoughts and emotions, knowing how to understand and sympathize with others’ thoughts, feelings, and perspectives
Care and respect	Acting in consideration of the position of others, understanding and respecting other positions, respecting the noble nature of life, being attentive to care for others, and caring for others
Honesty and humility	Being true or honest to yourself or others in a straightforward way, without lies or deception, without being arrogant or ignorant of others, knowing how to act in a humble way
Responsibility and calling	The intention of fulfilling one’s tasks faithfully and responsibly, protecting the basic rights and human rights of patients, appreciating the doctor’s profession, and contributing to society through profession
Collaboration and magnanimity	Attitude and ability to be interested in group and community issues, interacting with members and working together to achieve common goals
Creativity and positivity	Attitude of not being confined to existing frameworks, but being able to look at things and situations with new and open eyes, and seeking various ways to solve problems with good results even in difficult situations
Patience and leadership	Attitudes and ability to reflect on, examine, and endure in difficult situations, to view health care in its social context, and to reach agreement with other members of an organization
	
	

**Table 4.** Frequency of responses regarding the appropriate academic level for instruction on each core element

Core elements	Academic year								Total
	Premedical year				Medical year				
	Year 1	Year 2	Any	Year 1	Year 2	Year 3	Year 4	Any	
Service and sacrifice	2	-	31	5	3	4	4	19	68
Empathy and communication	-	2	28	6	2	5	4	21	68
Care and respect	1	-	26	4	5	5	8	18	67
Honesty and humility	3	1	25	2	1	4	6	24	66
Responsibility and calling	-	-	24	4	2	7	11	19	67
Collaboration and magnanimity	1	2	26	5	2	4	8	21	69
Creativity and positivity	2	-	29	6	4	2	3	22	68
Patience and leadership	1	3	22	3	1	6	10	18	64

Values are presented as number of respondents.

## Supplementary materials

Supplement 1. Data files are available from https://doi.org/10.7910/DVN/OQGBGC.

Supplement 2. Audio recording of the abstract.
