# Supplementary material for: Identification and evaluation of the core elements of character education for medical students in Korea
Source: J Educ Eval Health Prof. 2019 Aug 20;16:21. doi: 10.3352/jeehp.2019.16.21 (PMC6748878; doi:10.3352/jeehp.2019.16.21)
Supplement: Supplementary file 2 [file jeehp-16-21-app.pdf]

Appendix 1. The questionnaires of the Delphi survey

I. 연구대상자 설명서(델파이 조사)

연구과제명: 의과대학생의 인성교육을 위한 핵심요소 규명과 멘토링 모듈 개발

한국교육개발원의 조사(2014)에 따르면 국민의 72.4%는 우리나라 학생들의 인성·도덕성 수준이 전반적으로 낮으며, 인성교육이 가장 시급히 다뤄져야 할 교육문제라고 지적하고 있습니다. 따라서 본 연구는 우리나라 의과대학생의 인성교육을 위한 핵심요소를 규명하고 관련 모듈을 개발하는 연구입니다.

귀하는 본 연구에 참여할 것인지 여부를 결정하기 전에, 설명서와 동의서를 신중하게 읽어보셔야 합니다. 이 연구가 왜 수행되며, 무엇을 수행하는지 귀하가 이해하는 것이 중요합니다. 이 연구를 수행하는 **허예라 연구책임자**가 귀하에게 이 연구에 대해 설명해 줄 것입니다. 이 연구는 자발적으로 참여 의사를 밝히신 분에 한하여 수행될 것입니다. 다음 내용을 신중히 읽어보신 후 참여 의사를 밝혀 주시길 바라며, 만일 어떠한 질문이 있다면 담당 연구원이 자세하게 설명해 줄 것입니다.

귀하의 서명은 귀하가 본 연구에 대해 그리고 위험성에 대해 설명을 들었음을 의미하며, 이 문서에 대한 귀하의 서명은 귀하께서 자신이 본 연구에 참가를 원한다는 것을 의미합니다.

1. 연구의 배경과 목적

의사는 타 직종에 높은 소명의식과 윤리수준, 이타적인 가치관과 행동 등 일반적인 수준 이상의 인성이 요구되는 직종입니다. 의사 집단 그리고 의과대학생들의 인성과 관련한 사회적 문제가 끊임없이 발생하고 있는 현실은 현 의과대학 인성교육에 대한 점검과 새로운 인성교육의 필요성에 대한 공감대를 높입니다. 이에 이 연구는 의학교육에서의 인성교육을 재정의하고 의사가 갖추어야 할 인성의 핵심 요소를 규명하여 의과대학생을 대상으로 하는 인성교육의 졸업역량과, 시기별 학습성과, 교육내용과 방법에 대한 기준을 제시하고 의과대학 교육과정 안에서 실제로 적용할 수 있는 인성교육 멘토링 모듈(가칭)을 개발하는 것을 목표로 합니다

2. 연구 참여 대상

이 연구는 델파이 조사의 경우, 의학교육에 10년 이상 종사한 의학교육전문가들이 참여하게 되며, 참여자 수는 전국의대 40개 의과대학 의학교육분야 관련 분야에 종사하는 전문가 100명 내외로 예상하고 있습니다.

3. 연구방법

만일 귀하가 참여의사를 밝혀 주시면 다음과 같은 과정이 진행될 것입니다. 이 델파이 조사는 3회차 또는 필요한 경우 최대 4회차로 구분되어 실시되며 검사장소는 조사지가 우편으로 발송될 것이므로 각 연구대상자의 편리에 따른 자유로운 장소에서 실시하면 됩니다. 설문에 소요되는 시간은 평균 15분 내외로 예상되나, 특별히 정해진 시간은 없습니다.

설문방법은 먼저 개방형 질문을 통하여 우리나라 의사들에게 필요한 인성 요소들을 각 교육단계별로 추출하고, 추출된 의견을 기초로 편집한 구조화된 설문을 2-3차에 걸쳐 전문가들의 의견을 합의하는 과정이 반복될 것입니다. 따라서 1차 조사에 응답지를 회송하여 주셔야 2차 델파이 조사에 참여하실 수 있습니다.

교육과 연구 그리고 봉사활동으로 무척 바쁘실 것으로 사료되오나 교수님의 고견은 우리나라 의학교육에 필요한 인성교육의 요소를 규명하는 데에 매우 중요한 자료가 될 것이므로 귀중한 시간을 잠시 할애하여 주실 것을 부탁드립니다. 본 설문지에 작성한 내용은 철저하게 비밀이 보장되며 첨부된 질문지는 **이메일 회신 또는 회신용 봉투**를 사용하시어 **10월 5일까지** 회송하여 주시면 감사하겠습니다.

4. 연구 참여기간

귀하는 본 연구를 위해 델파이 조사결과 회송 여부에 따라 최대 3회차 또는 4회차까지 참여를 요청받으실 수 있습니다. 설문조사 기간은 2018년도 9-12월까지입니다.

5. 연구 참여 도중 중도탈락

귀하는 연구에 참여하신 후에도 언제든지 도중에 그만 둘 수 있습니다. 만일 귀하가 연구에 참여하는 것을 그만두고 싶다면 담당 연구원이나 연구책임자에게 즉시 말씀해 주십시오.

6. 연구 참여에 따른 이익

귀하가 이 연구에 참여하는 데 있어서 금전적 보상을 드리기는 어려우나, 귀하가 델파이 조사 마지막 회차까지 참여하실 경우 받는 제공하는 정보는 우리나라 의학교육 전문가들의 합의된 결과를 제공 받으시게 되며, 우리나라 의사에게 요구되는 인성 핵심요소가 규명되므로 의학교육 인성교육을 발전시키고 관련 내용에 대한 이해를 증진시키는 데에 큰 도움이 될 것입니다. 또한 규명된 핵심요소는 후차년도 연구를 통해 인성교육 모듈의 개발을 위한 기본자료로 활용될 것입니다.

7. 연구에 참여하지 않을 시 불이익

귀하는 본 연구에 참여하지 않을 자유가 있습니다. 또한 귀하가 본 연구에 참여하지 않아도 귀하에게는 어떠한 불이익도 없습니다.

8. 개인정보와 비밀보장

본 연구의 참여로 귀하에게서 수집되는 개인정보는 델파이 조사의 특성에 따라 기명 조사로 이루어집니다. 수집되는 정보는 성명, 소속대학, 의학교육분야 근무경력 기간, 전공, 직급, 주소와 연락처 정도입니다. 주소와 연락처의 경우 델파이 조사가 우편으로 시행되므로 발신, 회송용으로 불가피하게 필요합니다.

다만, 2회차 델파이 조사부터 공개되는 전문가들의 설문결과 내용에는 그 어떠한 개인정보도 공개되지 않습니다. 또한 수집되는 개인정보는 암호화되어 있는 파일에 보관되며 연구책임자와 보조연구자만 접근이 가능합니다.

연구를 통해 얻은 모든 개인 정보의 비밀보장을 위해 최선을 다할 것입니다. 이 연구에서 얻어진 개인 정보가 학회지나 학회에 공개될 때 귀하의 이름과 다른 개인 정보는 사용되지 않을 것입니다. 그러나 만일 법이 요구하면 귀하의 개인정보는 제공될 수도 있습니다. 또한 모니터 요원, 점검요원, 공용기관생명윤리위원회는 연구대상자의 비밀보장을 침해하지 않고 관련 규정이 정하는 범위 안에서 본 연구의 실시절차와 자료의 신뢰성을 검증하기 위해 연구결과를 직접 열람할 수 있습니다.

귀하가 본 동의서에 서명하는 것은 이러한 사항에 대하여 사전에 알고 있었으며 이를 허용한다는 의사로 간주될 것입니다. 연구 종료 후 연구 관련 자료는 5년간 보관되며 이후 서면 자료는 문서파쇄 기계를 통해, 컴퓨터 자료는 삭제 프로그램을 통해 폐기될 것입니다.

9. 연구 문의

본 연구에 대해 질문이 있거나 연구 중간에 문제가 생길 시 다음 연구 담당자에게 언제든지 연락하십시오(이름: 허예라; 전화번호: 010-0000-0000). 만일 어느 때라도 연구대상자로서 귀하의 권리에 대한 질문이 있다면 다음의 연구진이나 한림대학교 생명윤리위원회에 연락하십시오.

- 연구책임자 성명: 허예라
- 연구자 전화번호: 010-0000-0000 (24시간 연락처)
- 연구자 e-mail: shua@hallym.ac.kr

한림대학교 생명윤리위원회 전화번호 033-248-3021~2/e-mail: irb@hallym.ac.kr

II. 연구대상자 동의서(인간대상연구)

연구제목: 의과대학생의 인성교육을 위한 핵심요소 규명과 멘토링 모듈 개발

1. 나는 본 연구의 설명문를 읽었으며 담당 연구원과 이에 대하여 의논하였습니다.
2. 나는 위험과 이득에 관하여 들었으며 나의 질문에 만족할 만한 답변을 얻었습니다.
3. 나는 이 연구에 참여하는 것에 대하여 자발적으로 동의합니다.
4. 나는 이 연구에서 얻어진 나에 대한 정보를 현행 법률과 생명윤리심의위원회 규정이 허용하는 범위 내에서 연구자가 수집하고 처리하는데 동의합니다.
5. 나는 학교 당국 및 한림대학교 생명윤리위원회가 이 연구의 실시 절차와 자료의 신뢰성 확보를 위해 연구결과 및 동의서를 열람하는 것에 동의합니다.
6. 나는 언제라도 이 연구의 참여를 철회할 수 있고 이러한 결정이 나에게 어떠한 해도 되지 않을 것이라는 것을 압니다.
7. 나의 서명은 이 동의서의 사본을 받았다는 것을 뜻하며 연구 참여가 끝날 때까지 사본을 보관하겠습니다.

연구대상자	성명:		서명:		서명일:	
연구책임자	성명:		서명:		서명일:	

III. 우리나라 의과대학생의 인성교육을 위한 핵심요소 규명: 델파이 제1회차 설문

◎ 연구의 목적

이 연구는 의학교육에서의 인성교육을 재정의하고 의사가 갖추어야 할 인성의 핵심 요소를 규명하여 의과대학생을 대상으로 하는 인성교육의 졸업역량과, 시기별 학습성과, 교육내용과 방법에 대한 기준을 제시하고 의과대학 교육과정 안에서 실제로 적용할 수 있는 인성교육 멘토링 모듈(가칭)을 개발하는 것을 목표로 합니다.

◎ 용어의 정의

본 연구에서 의미하는 인성이란 좋은 의사가 되기 위해 갖추어야 할 윤리적/행동적 가치, 성품, 덕목, 인간상이라고 할 수 있습니다. 이는 좋은 의사에게 요구되는 지식, 기술, 태도를 모두 포함하는 포괄적인 개념의 의학전문직업성이나 의료인문학이라는 복합적인 개념과 겹치는 영역도 있겠으나, 동일하기보다는 구별된 차원으로 보는 것이 보다 바람직하겠습니다. 일부 선행연구에서는 인성을 성격(character)로 보는 견해도 있습니다. 의학교육계에서는 2014년 한국의 의사상 설정연구(보건복지부)를 통해 바람직한 의사의 역량을 '환자진료,' '소통과 협력,' '사회적 책무성,' '전문직업성' 및 '교육과 연구' 등 5가지 영역으로 규명하였고, 교육부의 2014년도 정책연구에서는 초, 중, 고등학교 인성교육의 핵심 요소를 자아정체감, 정직, 책임, 존중, 배려, 공감, 소통, 협동으로 발표한 바 있습니다.

◎ 질문지 응답방법

델파이 1차 설문의 가장 큰 목적은 개방형으로 형식의 제한 없이 응답자의 자유로운 의견을 많이 수집하는 것입니다. 이에 질문에 응답해 주실 때에는 가능한 구체적인 내용으로 풀어서 기술해 주시기 바랍니다.

예) 질문: '우리나라 의사에게 필요한 인성'

⇒ 응답 예: 타인을 존중하는 마음가짐, 타인을 위해 봉사하고자 하는 희생정신

성명		출생연도	년
학생 교육경력	년	의학교육 관련 부서 근무경력	년
		직장 근무경력	년
소속대학		직위	
소속부서(전공)		연락처(핸드폰)	

1. [인성교육의 필요성] 의학교육에서 인성교육이 필요하다고 생각하십니까?

2. [개념 정의] 의사에게 요구되는 인성적 소양 즉 "인성"은 무엇이라고 생각하십니까?

3. [문제점] 현재 의학교육이 갖고 있는 인성교육의 문제점이나 실패하고 있는 부분이 있다면 어떤 것이라고 생각하십니까?

4. 4차 산업시대의 의사가 갖추어야 할 인성의 핵심요소를 10가지 내외로 나열해 주시고, 중요도 순으로 1번부터 10번까지 번호로 매겨주십시오.

번호	요소	구체적인 의미	중요도(1-10위)
응답 예	봉사	타인을 위해 봉사하고자 하는 희생정신	2
1			
2			
3			
4			
5			
6			
7			
8			
9			
10			

※ 참여해 주셔서 감사합니다.

IV. 우리나라 의과대학생의 인성교육을 위한 핵심요소 규명: 델파이 제2회차 설문

델파이 2차 설문은 델파이 1차 설문의 결과를 토대로 작성되었습니다. 1차 설문결과 총 420개의 요소가 취합되었으며 연구팀은 (1) 유사한 개념을 가진 요소들을 묶어 총 17개의 항목으로 분류, (2) 빈도수에 근거하여 상위 10개의 항목을 선정한 후, (3) 이를 인성교육을 위한 핵심요소 “대분류명(가칭)”으로 규명하였습니다. 이때 대분류명은 항목 내에서 빈도수 1순위, 2순위 하위요소 용어를 기본으로 사용하여 명명한 후, 논의를 통해 하위요소를 대표하는 가장 적합한 용어로 최종 수정하였습니다.

◎ 질문지 응답방법:

1. [표1] 델파이 1차 결과로 추출된 요소에 대한 “교육시기,” “졸업생 수준,” 그리고 현재 대학에서 제공하고 있는 관련 요소의 교육정도에 대하여 만족도를 표기해주십시오.

2. 수정, 삭제, 추가 의견이 있으시면 [표2]를 활용해 주십시오.
① 수정할 “대분류명” 또는 “하위요소”를 적어주시고,
② □ 칸에 수정, 삭제, 추가 여부를 ✓표를 해 주시고
③ 수정 또는 추가 내용을 적어 주신 후
④ 수정, 삭제, 추가하신 이유(근거)를 적어 주시기 바랍니다.

3. 3차 델파이에서는 2차 설문에 대한 합의와 각 요소에 대한 교육방법과 평가방법에 대한 설문이 이루어질 계획입니다.

[척도 설명]

※ 교육 시기: [1] 1학년 [2] 2학년 [3] 3학년 [4] 4학년 [전] **전체 학년**

※ 졸업생 수준: ① 매우 낮다 ② 낮다 ③ 보통이다 ④ 높다 ⑤ 매우 높다

※ 교육정도: ① 매우 부족하다 ② 부족하다 ③ 보통이다 ④ 적절하다 ⑤ 매우 적절히 제공되고 있다

[표1] 좋은 의사가 갖추어야 할 인성 요소

순위	대분류명	하위요소(1차 응답 요소)	교육시기	졸업생 수준	관련 교육제공 정도(만족도)
1	봉사와 자율<br>Service & autonomy	봉사(24)/희생(8)/헌신/측은지심/연민/이타심/이타적 태도/따뜻함/의지/근면/양보/의지/박애/감사	의예과 [1] [2] [전]<br>의학과 [1] [2] [3] [4] [전]	① ② ③ ④ ⑤	① ② ③ ④ ⑤
2	소통과 공감<br>Communication & sympathy	의사소통능력(24)/공감(23)/소통지향성/갈등관리/경청/겸손(분류이동)/유연함/사교성/유머/건강한 대인관계/표현력/따뜻한 미소나 말투/자상함/화를 참는 마음/생각을 글과 말로 표현/인내	의예과 [1] [2] [전]<br>의학과 [1] [2] [3] [4] [전]	① ② ③ ④ ⑤	① ② ③ ④ ⑤
3	존중과 사랑<br>Respect & love	배려(25)/존중(12)/타인(환자포함)에 대한 이해/친절/관용/동반자의식/다양성에대한이해/사람에 대한 존중감/자기존중/자긍심/생명(인간)존중(5)/생명(인간)사랑(2)/생명윤리의식//죽음이해/인간성/예의	의예과 [1] [2] [전]<br>의학과 [1] [2] [3] [4] [전]	① ② ③ ④ ⑤	① ② ③ ④ ⑤
4	신뢰와 성실<br>Confidence & sincerity	정직(16)/성실(14)/겸손(7)/윤리적판단/도덕성/양심/도덕적 판단/준법정신/청렴성/진실/진정성/진지한마음/ 공정/정확성/성찰/원칙준수/ 지적 용기/ 직업윤리	의예과 [1] [2] [전]<br>의학과 [1] [2] [3] [4] [전]	① ② ③ ④ ⑤	① ② ③ ④ ⑤
5	책임과 의무<br>Responsibility & duty	책임감(22)/소명의식(3)/사명감/책무성/가치내면화/가치관/의료법/의료윤리/직업윤리/비밀유지/규칙준수/평생학습의 자세	의예과 [1] [2] [전]<br>의학과 [1] [2] [3] [4] [전]	① ② ③ ④ ⑤	① ② ③ ④ ⑤
6	협동과 포용<br>Cooperation/generosity	협동(12)/협력(7)/포용(3)/공동체의식/협동심/동료협업/팀워크/협조/교류/상호의존	의예과 [1] [2] [전]<br>의학과 [1] [2] [3] [4] [전]	① ② ③ ④ ⑤	① ② ③ ④ ⑤
7	창의적 사고와 비판적 사고<br>Creative & critical thinking	창의성(6)/긍정성(4)/통찰력/판단력/비판적 사고/결단력/개방적 사고/창의적 사고/다각도 상황을 바라보는 마음가짐/상상력/용기/침착/열정	의예과 [1] [2] [전]<br>의학과 [1] [2] [3] [4] [전]	① ② ③ ④ ⑤	① ② ③ ④ ⑤
8	리더십과 품격<br>Leadership & dignity	리더십(7)/도전정신(2)/팔로우십/선도/사회적 문제의식/사회적 인식능력/솔선수범/자기조절/관리/자기이해/자기반성/자기절제/자아정체감/역량파악/자제력/인내(10)/자기성찰(6)/자존감/건강/균형감/자기공감/예절/매너/예의/품위/품격/자기관리	의예과 [1] [2] [전]<br>의학과 [1] [2] [3] [4] [전]	① ② ③ ④ ⑤	① ② ③ ④ ⑤

[표2] 인성요소 분류표에 대한 의견란

대분류명/하위요소	수정방법(□표)과 이유
	□ 추가 □ 수정 □ 삭제<br>수정내용:<br>이유:
	□ 추가 □ 수정 □ 삭제<br>수정내용:<br>이유:
기타 의견:	

※ 협조해 주셔서 감사합니다.

V. 우리나라 의과대학생의 인성교육을 위한 핵심요소 규명: 델파이 제3회차 설문

델파이 3차 설문은 델파이 2차 설문의 결과를 토대로 작성되었습니다.

1) 2차 설문결과 1차 회신 총 47명 중 38명의 응답을 받았습니다. 이에 핵심요소에 대한 수정 의견들을 종합하여 재정리하였고, 대분류명은 크게 변동되지 않은 선에서 표현이 약간 수정되었습니다. 하위요소는 대분류명을 대표하는 3가지 요소로 요약 정리하였습니다.

2) 각 요소에 대한 적절한 교육시기는 의예과와 의학과 과정 중 어느 과정에서든지 개설이 가능하다는 의견이 많아 3회차 델파이 문항에서는 제외하였습니다. 따라서 3차 델파이에서는 졸업생 수준에 대한 의견 합의 문항만 포함하였습니다.

3) 3회차 델파이는 2회차 분석결과 응답분포도를 벗어나는 응답자에 한하여 실시됩니다.

◎ 응답방법: 2차 응답에 대한 합의

1. [표1]은 델파이 2차 결과로 분석된 응답 분포(음영 표기)와 귀하의 응답(*)을 분포도 위에 표기해두었습니다.
2. 귀하의 2차 응답이 분포도(음영 표기)를 벗어나는 항목에만 3차 응답을 해주시면 됩니다.
3. 응답 시 분포도 안으로 합의가 가능하신 경우 분포 안의 숫자에 표기를 해주시고, 동의하지 않을 경우 분포도 밖으로 표기하셔도 무방합니다.
4. 응답 예시가 3쪽에 있으니 참고하시기 바랍니다.

※ 응답회신은 **1월 28일(월)까지** 부탁드립니다.

※ 4차 델파이는 3차 설문에 합의가 되지 않는 문항이 나올 경우 실시됩니다.

[표1] 요소별 현재 졸업생 수준, 교육방법과 평가방법

척도설명
※ 현재 졸업생 수준<br>① 매우 낮다 ② 낮다 ③ 보통이다 ④ 높다 ⑤ 매우 높다<br><br>※ 교육방법<br>① 강의/특강 ② 현장경험학습 ③ 프로젝트 학습 ④ 역할극 ⑤ 사례분석 ⑥ 글쓰기 ⑦ 조사 ⑧ TBL (team based learning)<br>⑨ 토의/토론 수업 ⑩ PBL (problem based learning) ⑪ 기타 의견 (직접 써주시면 됩니다)<br><br>※ 평가방법<br>① 자가평가(설문지) ② 포트폴리오 ③ 표준화 환자 활용평가(OSCE, CPX 등) ④ MCQ 시험 ⑤ 보고서 ⑥ 기타 의견________________

※ 현재 졸업생 수준 응답은 귀하의 응답(*)이 응답분포를 벗어난 경우에만 해당되며, ✓표기 또는 해당 숫자를 다른 색으로 표기해주셔도 됩니다.

순위	대분류명	하위요소	현재 졸업생 수준<br>2회차 응답분포(음영 표기)<br>& 귀하의 응답 (*)<br>3회차 응답표기 (✓)	교육방법(위 척도에서 번호를 선택하세요. 중복선택 가능)	평가방법(위 척도에서 번호를 선택하세요. 중복선택 가능)
1	봉사와 희생	봉사, 성실, 희생, 이타심	(합의) *<br>1······2······3······4······5		
2	공감과 소통	의사소통능력, 대인관계, 표현력	(합의) *<br>1······2······3······4······5		
3	배려와 존중	존중, 배려, 친절	*<br>1······2······3······4······5		
4	정직과 겸손	정직, 겸손, 윤리의식	(합의) *<br>1······2······3······4······5		
5	책임과 소명	책임감, 소명의식, 가치관	(합의) *<br>1······2······3······4······5		
6	협동과 포용	협동, 포용력, 교류	(합의) *<br>1······2······3······4······5		
7	창의성과 긍정성	창의성, 긍정성, 개방적 사고	*<br>1······2······3······4······5		
8	인내와 리더십	인내, 자기성찰, 리더십	(합의)*<br>1······2······3······4······5		

[예시]

※ 졸업생 수준: 3번과 4번 문항만 합의가 필요하므로 2개 문항에만 □로 응답함

척도설명
※ 현재 졸업생 수준<br>① 매우 낮다 ② 낮다 ③ 보통이다 ④ 높다 ⑤ 매우 높다<br><br>※ 교육방법<br>① 강의/특강 ② 현장경험/실습 ③ 프로젝트 학습 ④ 역할극 ⑤ 사례분석 ⑥ 글쓰기 ⑦ TBL (team based learning) ⑧ 토의/토론 수업<br>⑨ PBL (problem based learning) ⑩ 기타 의견 (직접 써주시면 됩니다)<br><br>※ 평가방법<br>① 자가평가(설문지) ② 포트폴리오 ③ 표준화 환자 활용평가(OSCE, CPX 등) ④ MCQ 시험 ⑤ 보고서 ⑥ 기타 의견______

순위	대분류명	하위요소	현재 졸업생 수준<br>2회차 응답분포(음영 표기) & 귀하의 응답 (*) 3회차 응답표기 (✓)	교육방법(위 척도에서 번호를 선택하세요. 중복선택 가능)	평가방법(위 척도에서 번호를 선택하세요. 중복선택 가능)
1	봉사와 희생	봉사, 성실, 희생, 이타심	(합의) *<br>1······2······3······4······5	②, ⑤	②, ③, ⑤
2	공감과 소통	의사소통능력, 대인관계, 표현력	(합의) *<br>1······2······3······4······5	①, ④, ⑧	①, ③
3	배려와 존중	존중, 배려, 친절	* ✓<br>1······2······3······4······5	②, ④, ⑦	③
4	정직과 겸손	정직, 겸손, 윤리의식	(합의) * ✓<br>1······2······3······4······5	①, ⑤, ⑨	③, ④
5	책임과 소명	책임감, 소명의식, 가치관	(합의) *<br>1······2······3······4······5	③, ⑥, ⑧	②

6	협동과 포용	협동, 포용력, 교류	(합의) * 1······2······3······4······5	① , ③, 개별발표	②, ⑤
7	창의성과 긍정성	창의성, 긍정성, 개방적 사고	(합의) * 1······2······3······4······5	③ , ⑦ , 조별발표	① , 동료평가
8	인내와 리더십	인내, 자기성찰, 리더십	(합의) * 1······2······3······4······5	②, ③, ⑤, ⑥	②, ⑤
